# Deletion of the entire interferon-γ receptor 1 gene causing complete deficiency in three related patients

**DOI:** 10.1007/s10875-016-0244-y

**Published:** 2016-03-01

**Authors:** Inge C. de Vor, Pomme M. van der Meulen, Vincent Bekker, Els M. Verhard, Martijn H. Breuning, Esther Harnisch, Maarten J. D. van Tol, Jantien W. Wieringa, Esther van de Vosse, Robbert G. M. Bredius

**Affiliations:** Department of Pediatrics, Leiden University Medical Center, Albinusdreef 2, 2333 ZA Leiden, The Netherlands; Department of Pediatrics, Medical Center Haaglanden, Lijnbaan 32, 2512 VA The Hague, The Netherlands; Department of Infectious Diseases, Leiden University Medical Center, Albinusdreef 2, 2333 ZA Leiden, The Netherlands; Department of Clinical Genetics, Leiden University Medical Center, Albinusdreef 2, 2333 ZA Leiden, The Netherlands

**Keywords:** MSMD, IFN-γR1 deficiency, *IFNGR1*, *Mycobacterium fortuitum*, EBV, *IL22RA2*

## Abstract

**Purpose:**

Complete interferon-γ receptor 1 (IFN-γR1) deficiency is a primary immunodeficiency causing predisposition to severe infection due to intracellular pathogens. Only 36 cases have been reported worldwide. The purpose of this article is to describe a large novel deletion found in 3 related cases, which resulted in the complete removal of the *IFNGR1* gene.

**Methods:**

Whole blood from three patients was stimulated with lipopolysaccharide (LPS) and IFN-γ to determine production of tumor necrosis factor (TNF), interleukin-12 p40 (IL-12p40) and IL-10. Expression of IFN-γR1 on the cell membrane of patients’ monocytes was assessed using flow cytometry. *IFNGR1* transcript was analyzed in RNA and the gene and adjacent regions were analyzed in DNA. Finally, *IL22RA2* transcript levels were analyzed in whole blood cells and dendritic cells.

**Results:**

There was no expression of the IFN-γR1 on the monocytes. Consistent with this finding, there was no IFN-γ response in the whole blood assay as measured by effect on LPS-induced IL-12p40, TNF and IL-10 production. A 119.227 nt homozygous deletion on chromosome 6q23.3 was identified, removing the *IFNGR1* gene completely and ending 117 nt upstream of the transcription start of the *IL22RA2* gene. Transcript levels of *IL22RA2* were similar in patient and control.

**Conclusions:**

We identified the first large genomic deletion of *IFNGR1* causing complete IFN-γR1 deficiency. Despite the deletion ending very close to the *IL22RA2* gene, it does not appear to affect *IL22RA2* transcription and, therefore, may not have any additional clinical consequence.

**Electronic supplementary material:**

The online version of this article (doi:10.1007/s10875-016-0244-y) contains supplementary material, which is available to authorized users.

## Introduction

Complete interferon-γ receptor 1 (IFN-γR1) deficiency is an autosomal recessively inherited immunodeficiency, characterized by predisposition to infections with intracellular pathogens, in particular mycobacteria. This rare genetic defect disrupts the interferon-γ (IFN-γ) pathway, leading to one of the 19 genetic etiologies of Mendelian Susceptibility to Mycobacterial Diseases (MSMD) [1–3]. Complete IFN-γR1and complete IFN-γR2 deficiency, represent the most severe phenotypes of MSMD, whereas partial IFN-γR1 deficiency is associated with a later onset and milder disease course.

Most commonly, patients with complete IFN-γR1 deficiency present with lymphadenopathy, hepatosplenomegaly and intermittent fever in early childhood, caused by infection with weakly virulent, mostly environmental mycobacteria such as *Mycobacterium avium* or the vaccine strain *Mycobacterium bovis* bacillus Calmette-Guérin (BCG) [2]. Furthermore, patients with a complete defect appear to be prone to develop malignancies [4–6]. Hematopoietic stem cell transplantation (HSCT) is required to restore normal immune function. Unfortunately graft failure rates are high [7, 8] and consequently, the overall prognosis of patients with complete IFN-γR1 deficiency remains poor.

The IFN-γR1 gene (*IFNGR1*) is located on chromosome 6q23.3 and stretches over 22 kb. Twenty-seven unique mutations causing complete IFN-γR1 deficiency have been identified so far. These are all small variations, with the largest deletion being only 22 nucleotides long (see Table 1). This is the first description of a large genomic deletion, removing *IFNGR1* entirely and causing complete IFN-γR1 deficiency in three related patients.

**Table 1:** Summary of patients with complete IFN-γR1 deficiency

Patient/ kindred	cDNA change^a,b^	Sex	Country	Significant pathogens	HSCT	Graft failure	Status at time of publication	Age (year)^c^	Other pathology	Ref
With HSCT										
1a	105dup	F	Turkey	MAC, *M. tuberculosis*, *S. enteritidis*	Yes	No^d^	Alive 4 years after HSCT, infection free	9		[3, 7]
2a	105dup	M	Turkey	–	Yes	No	Died 7 weeks after HSCT of hepatic bleeding on biopsy	5^f^		[3, 7]
3b	105dup	M	Turkey	BCG, MTC, MAI, *M. bovis, M. fortuitum*, VZV, *P. aeruginosa*	Yes	No	Died 9 months after HSCT of disseminated infection	6^f^		[9]
4c	182 T → A / 652del3	F	France	BCG	Yes, twice	1: Yes	Died 7 months after HSCT of EBV-LPS	5^f^	Grade III GvHD	[3, 7, 10]
						2: No				
5d	201-2A → G	M	Pakistan	MAC, CMV, PIV-4, RSV	Yes, twice	1: Yes	Died 1 year after HSCT of disseminated infection	5^f^	Grade III GvHD	[3, 7, 11–13]
						2: No				
6e	230G → T	M	Unknown	*M. fortuitum*	Yes	No	Alive 2 years after HSCT, infection free	4		[14]
7f	295_306del	F	Algeria	BCG, MAC	Yes	No	Died 2 months after HSCT of granulomas with MAC	3^f^		[3, 7, 10]
8g	347C → A	M	Malta	MAC	Yes	No	Alive 6 years after HSCT, infection free	14	VOD	[3, 7, 15, 16]
9h	373 + 1G → T / 563_566del	F	Germany	BCG, *M. kansasii,,* MAC*,* VZV, *L. monocytogenes*	Yes	No	Alive 6 years after HSCT, infection free	14	Severe liver cirrhosis	[3, 7, 17, 18]
10i	523delT	M	Italy	*M. peregrinum*	Yes, twice	1: Yes	Alive 21 months after 2nd HSCT, persistent disease	6		[3, 7, 19]
						2: Yes				
11j	523delT / 653_655del	M	Spain	*M. fortuitum, Salmonella sp.*	Yes	No	Alive 13 months after HSCT, infection free	5		[20]
Without HSCT										
12k	25del	M	Pakistan	MAC, *M. abscessus*	No		Died of B-cell lymphoma	20^f^	B-cell lymphoma	[3, 6, 12, 21, 22]
13k	25del	M	Pakistan	BCG, MAC, *E. histolytica*	No		Died of disseminated infection	6^f^		[12, 21]
14l	104_107dup / 200 + 1G → A	F	Italy	*M. smegmatis*	No		Died, cause not specified	7^f^		[3, 23, 24]
15m	106_107insT / 197A → G	M	Greece	*Mycobacterium sp., Salmonella sp.*	No		Died of disseminated Salmonellosis	1^f^		[3]
16m	106_107insT / 197A → G	M	Greece	*M. fortuitum*	No		Died, cause not specified	5^f^		[3]
17n	114_135del	F	China	BCG	Unknown		Alive, status unknown	2		[25]
18o	131delC	F	Tunisia	BCG	No		Died of disseminated infection	0^f^		[3, 26]
19p	166del	M	Canada	BCG, CMV, *M. bovis*	No		Died of disseminated infection	0^f^		[3, 27]
20q	170del	M	USA	MAC	Unknown		Alive, status unknown	4		[3]
21r	230G → A	M	Turkey	BCG, HHV-8, *M. fortuitum*	No		Died of Kaposi sarcoma and disseminated infection	12^f^	Kaposi sarcoma	[3, 4, 10]
22r	230G → A	F	Turkey	BCG, *M. fortuitum*, *M. tuberculosis*	No		Died of disseminated infection	13^f^		[3, 4, 10]
23s	254G → A	F	Pakistan	BCG, *M. avium*	No		Died of disseminated infection	1^‡^		[28, 29]
24t	339 T → A	M	Italy	*M. fortuitum, R. equi*	Unknown		Alive with complex syndrome, free of mycobacterial infection	8	UPD chr. 6	[30]
25g	347C → A	M	Malta	*M. chelonei*	No		Died of progressive pneumonia	3^f^		[3, 15, 16]
26g	347C → A	M	Malta	*M. fortuitum*	No		Died of disseminated infection	9^f^		[3, 15, 16]
27u	347C → A	F	Malta	MAI	No		Died of disseminated infection	6^‡^		[3, 15, 16]
28v	373 + 1G → T	M	Netherlands	*M. gordonae, M. peregrinum, M. mageritense, M. szulgai, M. scrofulaceum, E. faecalis*	Unknown		Alive and B cell lymphoma in complete remission	34	Cholestatic liver disease, thrombo-embolisms, B-cell lymphoma	[29]^e^
29w	373 + 1G → T	M	Netherlands	*M. tilburgii*	Unknown		Alive, infection free	5		[31]
30x	453delT	M	Greece	*M. fortuitum – M. peregrinum* complex	Unknown		Alive, active infection	4		[32]
31y	523delT	M	Greece	BCG	Unknown		Alive, status unknown	15		[3]
32z	523delT	M	Italy	*M. scrofulaceum*	Unknown		Alive and 4 years tumor-free	15	Pineal germinoma	[5, 33]
33aa	563_566del	F	Argentina	BCG, MAC	Unknown		Alive, status unknown	4		[3, 34, 35]
34bb	655G → A	F	Chinese	*M. tuberculosis,* EBV	No		Died of disseminated infection	4^f^	HLH	[36]
35cc	683delC	F	Dominican Republic	MAC	Unknown		Alive, active infection	4		[37]
36dd	1454C → T	F	Egypt	BCG	No		Died, cause not specified	16^f^		[38]
The reported patients										
Case 1	Complete deletion	F	Turkey	*M. fortuitum*	No		Alive, infection free	1		–
Case 2	Complete deletion	F	Turkey	Unknown	No		Alive, elevated inflammatory parameters	4		–
Case 3	Complete deletion	F	Turkey	EBV	No		Alive, infection free	2		–

*MTC M. tuberculosis complex MAI M. avium intracellulare, MAC M. avium complex, BCG M. bovis* bacillus Calmette-Guérin, *CMV* cytomegalovirus, *EBV* Epstein-Barr virus, *VZV* varicella-zoster virus, *HHV-8* human herpesvirus 8, *RSV* respiratory syncytial virus, *PIV-3* parainfluenza virus type 3, *S. enteriditis, Salmonella enteriditis, P. aeruginosa Pseudomonas aeruginosa, E. histolytica Entamoeba histolytica, R. equi Rhodococcus equi, L. monocytogenes Listeria monocytogenes, C. jejuni Campylobacter jejuni, E. faecalis Enterococcus faecalis, VOD* veno-occlusive disease, *EBV- LPS* Epstein-Barr virus associated lymphoproliferative syndrome, *UPD chr. 6* uniparental disomy chromosome 6

^a^Homozygous unless otherwise specified

^b^Official terminology of mutation

^c^Age in years at death or last follow-up

^d^2 % chimerism at last follow-up

^e^Unpublished data

^f^Deceased

## Case Reports

**Fig. 1:**
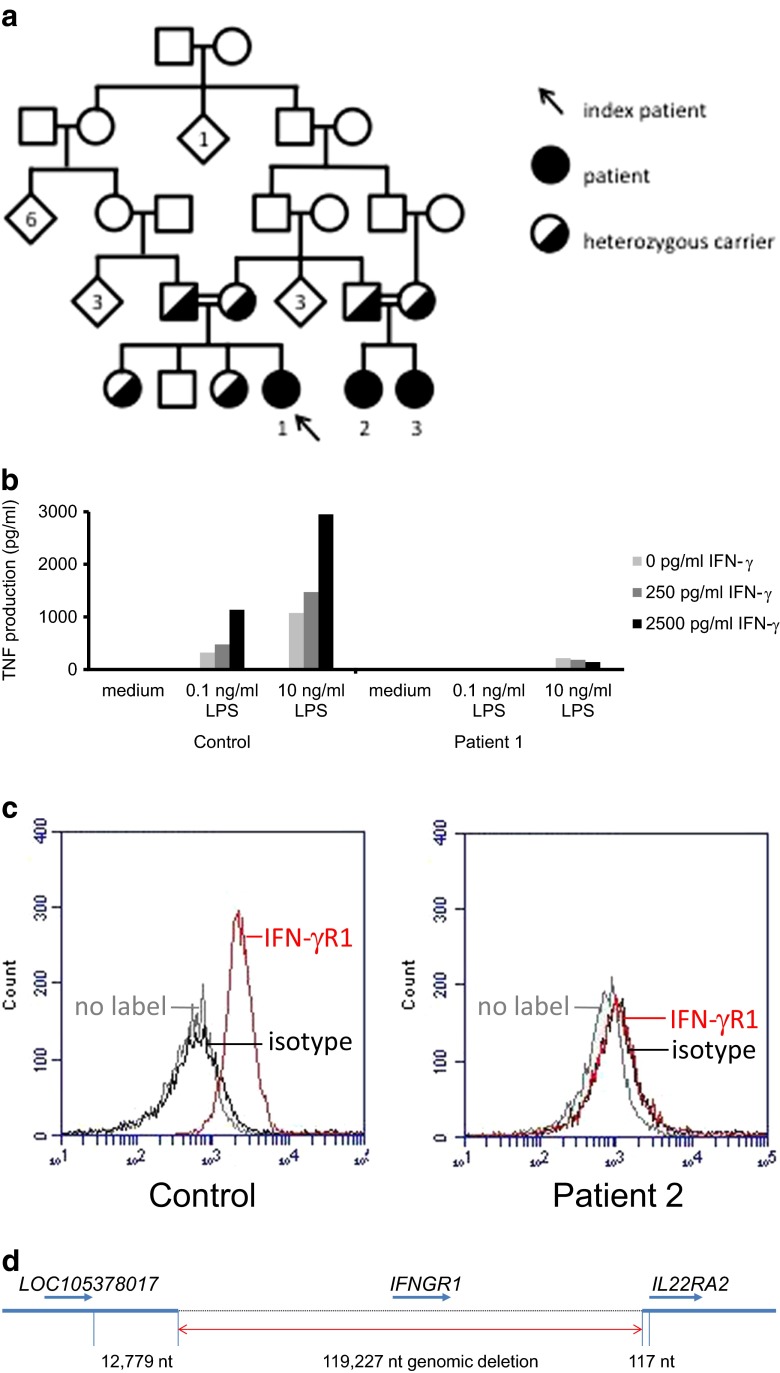
Pedigree, immunological assays and genetic analysis of patients. Family tree of patients *1*, *2* and *3* (**a**). In vitro TNF production in response to stimulation with LPS plus various concentrations of IFN-γ in patient 1 and healthy control (**b**). Flow cytometry showing absent cell surface expression of IFN-γR1 (GIR-94 antibody, BD Biosciences) on monocytes of patient 2 (**c**). Large homozygous deletion on chromosome 6q23.3 identified with PCR and sequencing, removing the entire IFN-γR1 gene (*IFNGR1*) and surrounding region (**d**). Deletion terminates 117 nt upstream of the transcription start of *IL22RA2* (not to scale). The first and last nucleotides of the deletion are: 137,173,766 and 137,292,992 *H. sapiens* chromosome 6, GRCh38.p2 Primary Assembly

A 1-year-old girl (patient 1) of Turkish origin was seen in the outpatient department with unilateral cervical lymphadenitis, existing for 1 month despite treatment with flucloxacillin by her family doctor. Apart from a persisting rhinitis the child had no other complaints, especially no fever, night sweats, weight loss, orthopnea or signs of hemorrhagic diathesis. There was no history of animal contact or visits to foreign countries. Her medical history included two episodes of respiratory tract infections at the age of 6 and 7 months, requiring admission to the hospital. Oxygen therapy, oral macrolide antibiotics and bronchodilators were given. Chest X-rays showed bilateral consolidations during the first admission, which were resolved a month later. Beclomethasone inhalation therapy was started after discharge. She was vaccinated according to the Dutch national program, which does not include BCG vaccine. Parents were consanguineous (Fig. 1a), but otherwise the family medical history was unremarkable.

Physical examination revealed multiple small cervical lymph nodes and one enlarged left pre-sternocleidomastoid node (4 × 2 cm) without fluctuation or redness of the overlying skin. Laboratory analysis revealed a leukocytosis with increased granulocyte and lymphocyte numbers without leukemic blasts, a mild anemia and a normal thrombocyte count. Chest X-ray was normal. Serologic tests for streptococcus, Bartonella sp., toxoplasmosis, Epstein-Barr virus and cytomegalovirus were negative. A bacterial infection was suspected and amoxicillin/clavulanic acid was administered.

In light of progressive enlargement of the cervical lymph node (4 × 6 cm) and appearance of supraclavicular nodes in the next weeks, other diagnoses, such as malignancy and (atypical) mycobacterial infection were considered. Screening for anti-nuclear antibodies, sarcoidosis, human immunodeficiency virus, germ cell tumor and neuroblastoma was negative. Ultrasound did not show abscess formation or intra-abdominal lymphadenopathy. Quantitative immunoglobulin levels revealed marginally raised IgM and IgA levels, whereas IgG level was normal. Peripheral blood T-lymphocyte counts were performed and showed increased CD3 and CD4 counts and CD4 effector-memory population, whereas the naïve T-cells were mildly decreased. The tuberculin skin test showed an induration of 7.0 mm. The result of the IFN-γ release assay (QuantiFERON®) showed high values for the specific mycobacterial antigens, however, the assay was interpreted as invalid because of very high IFN-γ values obtained for the negative control (64 IU/ml, normal <0.35 IU/ml). The high IFN-γ in the negative control can however be indicative of a complete IFN-γR defect. Fine needle aspiration of the cervical lymph node showed non-specific inflammation. Hence, bone marrow aspiration and lymph node excision were performed. Apart from non-specific reactive inflammation of the lymph node, no histologic abnormalities, especially no granuloma formation or malignancy, were noted. Bone marrow examinations revealed reactive changes only with no evidence of malignant infiltration. After 2 weeks lymph node cultures became positive for *Mycobacterium fortuitum*. Treatment was started with ciprofloxacin and co-trimoxazole, eventually resulting in almost complete normalization of all laboratory parameters. The negative control in the QuantiFERON® test decreased to 2.6 IU/ml (normal <0.35 IU/ml). Importantly, the clinical condition of the patient gradually improved.

Several weeks later the 3.5-year-old and almost 2-year-old female cousins (patient 2 and 3, respectively) presented with bilateral cervical lymphadenitis, which had been evident for almost 2 weeks. Accompanying symptoms were rhinitis, cervical pain and low-grade fever, which resolved spontaneously. Apart from one episode of bronchial hyper-reactivity (patient 2) and rotavirus infection (patient 3) their medical history was unremarkable. No BCG vaccine had been administered. Their parents were first cousins and from the same consanguineous Turkish family (Fig. 1a).

Physical examination revealed hepatosplenomegaly and bilateral enlarged lymph nodes (patient 2: mid-jugular left side 3.0 × 1.5 cm and right side 3.5 × 3.0 cm; patient 3: upper-jugular left side 3.0 × 2.0 cm and right side 2.0 × 1.0) without fluctuation or redness. Laboratory analyses showed elevated inflammatory parameters (ESR, CRP and leukocytosis) and anemia in patient 2. Peripheral blood T-lymphocyte counts showed increased CD3 and CD4 effector-memory population in patient 2 and an increased CD8 effector-memory population in patient 3. Plasma EBV DNA analysis and EBV serology indicated primary EBV infection in both patients (*i.e.* patient 2: IgG EBV-VCA and IgG EBV-EBNA positive; patient 3: EBV PCR 300 copies/ml at admission becoming negative in association with seroconversion of IgM to IgG EBV-VCA). QuantiFERON® test showed similar results with high negative control values in the IFN-γ release assay as in patient 1. Ultrasound analysis confirmed the presence of hepatosplenomegaly, without intra-abdominal lymphadenopathy nor abscess formation of the cervical nodes. Fine needle aspiration of the cervical lymph nodes showed no mycobacterial infection or other abnormalities.

Patients 2 and 3 were closely monitored without prophylaxis for mycobacterial diseases. During this follow-up a skin infection developed in patient 2 due to varicella zoster virus and in patient 3 due to *Staphylococcus aureus*, which were successfully treated with valaciclovir and flucloxacillin, respectively. The treatment of patient 3 required two days hospitalization for intravenous flucloxacillin. Otherwise the clinical courses were unremarkable. Although the size of the lymph nodes decreased over time in both patients, the hepatosplenomegaly and inflammatory parameters were persistently elevated for several months in the eldest (patient 2) in contrast to her younger sister (patient 3), in whom spontaneous resolution was observed over a period of 4 weeks. Because of the persisting lymphadenopathy in patient 2, bone marrow aspiration and lymph node excision were recently performed. Very similar to the observation in patient 1, non-specific reactive inflammation of the lymph node and no histologic abnormalities, especially no granuloma formation or malignancy, were noted. EBV in situ hybridization was negative, and no other (mycobacterial) infection could be detected.

IFN-γ concentrations were determined in serum samples of the patients by Luminex assay. These were on the day of their first visit our clinic 2330, 1144 and 1568 pg/ml (normal 7–124 pg/ml) in patient 1, 2 and 3, respectively. During follow-up (after 6.5, 3.5 and 3.5 months) these IFN-γ concentrations decreased to 298, 707 and 428 pg/ml in patient 1, 2 and 3, respectively.

## Materials and Methods

### Immunological Assays

Whole blood assay and IFN-γR1 expression analysis were performed as previously described [39, 40].

### Genetic Analysis of *IFNGR1*

DNA and RNA were isolated from whole blood; cDNA was synthesized from RNA with SuperScript III (Invitrogen, Bleiswijk, the Netherlands). Reverse transcription polymerase chain reaction (RT-PCR) of the *IFNGR1* transcript from cDNA was performed (primers and conditions available on request). PCRs were performed to amplify all exons of *IFNGR1* from genomic DNA. Various primer sets were subsequently designed to amplify the genomic region around *IFNGR1* and to determine the extent of the deletion. The primers that were found to cover the deletion were DEL307F 5′-AAAGCTTGGTTTCATGCTCTAA-3′ and DEL307R 5′-GGGACGCCATGTTATGTTTT-3′. These are located at 137293106–137293085 and 137173480–137173499, respectively, on *Homo sapiens* chromosome 6, GRCh38.-2 Primary Assembly.

### Analysis of *IL22RA2* Transcription by Dendritic Cells and Whole Blood Cells

PBMCs isolated from whole blood were cultured in IMDM (containing 10 % human serum, glutamine, penicillin, streptomycin) allowing monocytes to adhere to the flask surface. Non-adherent cells were washed away after 18 h. Monocytes were cultured for 4 days with 20 ng/ml GM-CSF (Sanquin, Amsterdam, NL) and 20 ng/ml IL-4 (Peprotech, London, UK) to induce differentiation to dendritic cells and 100 nM retinoid derivative AM580 (Sigma, St. Louis, MI, USA) to induce *IL22RA2* transcription. Cells were washed and cultured for another 3 days in IMDM containing 10 % fetal calf serum, glutamine, penicillin, streptomycin, GM-CSF, IL-4 and AM580. RNA was isolated, cDNA was synthesized with SuperScript III (Invitrogen) and qRT-PCR was performed to detect *TBP* (reference gene) and *IL22RA2* transcripts (primers and conditions available on request). qRT-PCR was also performed in RNA isolated from whole blood cells.

## Results

### No IFN-γ Response in patients’ Cells and no IFN-γR1 Expression on patients’ Monocytes

To determine whether the IFN-γ pathway was functional, we stimulated whole blood of patient 1 with LPS and IFN-γ. TNF production was low in response to LPS and not upregulated by the addition of IFN-γ in various concentrations (Fig. 1b). Furthermore, there was no upregulation of IL-12p40 or downregulation of IL-10 in response to the addition of IFN-γ (data not shown). The patient is capable of producing cytokines in the same whole blood assay, as illustrated by the IL-10 production in response to LPS (Supplemental Figure 1). Results of patients 2 and 3 were identical. Flow cytometry showed that cell surface expression of IFN-γR1 on patients’ monocytes was absent (Fig. 1c).

### The Patients are Homozygous for a Large Deletion Removing *IFNGR1* Completely

Subsequent analysis of RNA of patient 1 showed that *IFNGR1* transcripts were absent and *IFNGR1* exons could not be amplified from genomic DNA of the patient. Using various primer combinations in the regions flanking the *IFNGR1* gene, we were able to establish that a genomic deletion of 119.227 nt was present (Fig. 1d). The first and last nucleotides of the deletion are 137173766 and 137292992 (*Homo sapiens* chromosome 6, GRCh38.p2 Primary Assembly) encompassing the entire *IFNGR1* gene but leaving the flanking genes *LOC105378017* (uncharacterized gene) and *IL22RA2* (encoding a soluble IL-22 receptor, also known as IL-22BP) intact. The deletion was homozygously present in all three patients and heterozygously in all four parents and two of the three siblings of patient 1 (Fig. 1a).

### Similar *IL22RA2* Transcript Levels in Patient 2 and Control

Because the genomic deletion ended only 117 nt upstream of the transcription start of *IL22RA2* we hypothesized this deletion could affect its transcription. Therefore, we analyzed *IL22RA2* transcripts by RT-PCR in RNA isolated from dendritic cells stimulated with the retinoid derivative AM580, which is known to upregulate *IL22RA2* transcription. In both patient 2 and a control *IL22RA2* transcript variant 2 (lacking exon 4, containing exon 6) was the most abundant transcript, while *IL22RA2* transcript variant 1 (containing all exons) was also present. *IL22RA2* transcript variant 3 (lacking exon 4 and 6) was not detected in the samples. To determine whether the promoter is not continuously active we assessed whether IL22RA2 transcripts were detectable in whole blood cells. In RNA isolated from whole blood cells *IL22RA2* transcripts were not detectable in samples from the three patients and a control (data not shown). qRT-PCR for *IL22RA2* showed that the ratio between patient/control transcript levels is 1,92 in the AM580-stimulated dendritic cells and confirmed that in whole blood *IL22RA2* transcripts are undetectable in the patient and control.

## Discussion

We describe three related patients with complete IFN-γR1 deficiency. They presented between 1 and 4 years of age with persistent or marked cervical lymphadenopathy as the main symptom. Lymph node tissue from patient 1 cultured *M. fortuitum* as the causative pathogen and primary EBV infection was found in patients 2 and 3.

To date, only 36 cases of complete IFN-γR1 deficiency have been reported. These patients are summarized in Table 1. All but one patient developed mycobacterial infection, including BCG post vaccination infections in fourteen cases. In addition, other significant pathogens were identified in approximately one third of the cases. The disease course of these infections was either comparable (*e.g.* EBV) or more severe (*e.g. Salmonellae, Pseudomonas aeruginosa, Listeria monocytogenes*) than observed in immunocompetent subjects. Furthermore, late-onset malignancy (pineal germinoma, Kaposi sarcoma and two cases of B-cell lymphoma) following diagnosis developed in four cases (Table 1: patients 32z, 21r, 12 k and 28v, respectively).

The clinical presentation of patient 1 is similar to previously reported cases. *M. fortuitum* has been identified in eight previous cases, and is therefore a well-known pathogen causing disease in patients with complete IFN-γR1 deficiency (Table 1). Furthermore, the very high serum levels of IFN-γ as detected in the negative control of the Quantiferon test and in the Luminex assay are correlated with the lack of IFN-γR1 expression on the cell surface. This phenomenon has been described before in patients with complete IFN-γR1 or IFN-γR2 deficiency [41]. Patients 2 and 3 presented with cervical lymphadenopathy and splenomegaly due to primary EBV infection, which has not been reported before as a first presentation in patients with complete IFN-γR1 deficiency. The clinical disease course of the EBV infection was relatively unremarkable, but hepatosplenomegaly and inflammatory parameters (*i.e.* elevated ESR, CRP, leukocytosis, lymphocytosis, and hypergammaglobulinemia) were more pronounced compared to EBV infection in subjects with normal immune function. In patient 2 these laboratory parameters remained abnormal for over 4 months. When compared to the previously described cases this benign clinical phenotype is likely due to lack of exposure thus far in these very young patients to pathogenic mycobacteria.

Twenty-seven different mutations causing complete IFN-γR1 deficiency have been reported (Leiden Open Variation Database, www.lovd.nl/IFNGR1 and Table 1). These mutations are single nucleotide variations, small duplications, insertions or deletions. The largest reported genomic deletion was only 22 nt long (patient 17n, Table 1). Complete absence of *IFNGR1* due to a large deletion has not been reported previously. It remains unclear whether the size of the deletion (119.227 nt) has additional clinical consequences other than complete IFN-γR1 deficiency. No coding regions besides *IFNGR1* are known to be located in the area of the deletion. However, the deletion terminates 117 nt upstream of the transcription start of the *IL22RA2* gene, raising suspicion that binding of transcription-regulating factors might be affected. Transcription of *IL22RA2* leads to production of IL-22 binding protein (IL-22BP), a soluble receptor, which is capable of binding and inactivating IL-22 [42]. IL-22BP is produced by dendritic cells [43]. We were able to detect *IL22RA2* transcription in response to retinoid stimulation of the dendritic cells that was similar between patient and control in both abundance and transcript variants present. Transcript variant 2, encoding the IL-22BP isoform which efficiently binds and inhibits IL-22 [42, 44], was the most abundant transcript detected. To determine whether the promoter is not continuously active we also assessed whether *IL22RA2* transcripts were detectable in whole blood cells. This was neither the case in patients nor in control RNA. Together these results suggest that *IL22RA2* transcription is not affected.

At time of publication, infectious parameters of patient 2 remain elevated several months after primary EBV infection, while bacterial cultures of lymph node and blood are repeatedly negative. Patients 1 and 3 are in good clinical condition, without signs of active infection. Hematopoietic stem cell donor searches are in progress in order to facilitate future HSCT for all three children. Unfortunately, graft failure has been reported in approximately one third of transplanted cases (Table 1). This increased rate is most likely due to high plasma concentrations of IFN-γ [41], which has anti-hematopoietic activity [45]. Options for reducing plasma IFN-γ around HSCT with anti-IFN-γ monoclonal antibodies are currently being explored.

In conclusion, we report three related cases of complete IFN-γR1 deficiency caused by a novel large genomic deletion, removing *IFNGR1* entirely and ending close to the *IL22RA2* gene. The disease course of the patients reported here was relatively unremarkable and similar to previously reported cases of complete IFN-γR1 deficiency, except for primary EBV infection as the presenting infection in two of three patients.

## Electronic supplementary material

Supplemental Figure 1 (DOCX 50 kb)
