# Supplementary material for: Deletion of the entire interferon-γ receptor 1 gene causing complete deficiency in three related patients
Source: J Clin Immunol. 2016 Mar 1;36:195–203. doi: 10.1007/s10875-016-0244-y (PMC4792359; doi:10.1007/s10875-016-0244-y)
Supplement: Supplementary file 1 — (DOCX 50 kb) [file 10875_2016_244_MOESM1_ESM.docx]

**Supplemental Figure 1**

**Legend to Supplemental Figure 1**

In vitro IL-10 production in response to stimulation with various concentrations of LPS in patient 1 and healthy control, as measured in the same whole blood assay as Figure 1b.
